# Effects of irritable bowel syndrome and related cognitive-behavioral and occupational stress factors on the productivity and abdominal symptoms of Japanese workers: a longitudinal study

**DOI:** 10.1186/s13030-026-00349-z

**Published:** 2026-01-19

**Authors:** Nagisa Sugaya, Shuhei Izawa, Takeshi Sasaki

**Affiliations:** Occupational Stress and Health Management Research Group, National Institute of Occupational Safety and Health, 6-21-1 Nagao, Tama-ku, Kawasaki, Kanagawa 214-8585 Japan

**Keywords:** Abdominal symptoms, Cognitive-behavioral factors, Irritable bowel syndrome, Psychosocial characteristics, Worker, Work productivity

## Abstract

**Objectives:**

This longitudinal study aimed to examine the effects of irritable bowel syndrome (IBS) and related cognitive-behavioral and occupational stress factors on the productivity and abdominal symptoms of Japanese workers.

**Methods:**

An online survey was conducted from October 5 to October 20, 2023 (Time 1), targeting workers aged 20–49 years who were not self-employed or business owners and who worked more than 30 h per week. Data from 1,062 participants was available. A follow-up survey was conducted one year later (Time 2), with responses from 424 participants (161 women and 114 persons who reported IBS in the Time 1 screening) used for the analysis.

**Results:**

Hierarchical multiple regression analysis showed that depression, physical symptoms, and the presence of IBS at Time 1 had significant effects on presenteeism and overall work productivity impairment at Time 2. In the IBS group, the interaction between maladaptive cognition related to IBS and job control at Time 1 were significantly related to abdominal symptoms at Time 2, indicating that IBS symptoms were more severe when job control was low and maladaptive cognition related to IBS was high, although the simple slope test results did not reach statistical significance. The physical symptoms of the IBS group at Time 1 had significant effects on presenteeism and overall work productivity impairment at Time 2; however, occupational stress factors and IBS-related cognitive-behavioral variables did not show significant effects.

**Conclusion:**

The presence of IBS was shown to be related to the magnitude of productivity impairment one year later. Additionally, a correlation was seen between job control and maladaptive cognition regarding the effect of IBS on abdominal symptoms up to one year later. However, no evidence was found that occupational stress factors or IBS-related cognitive-behavioral variables were related to work productivity one year later.

## Introduction

Irritable bowel syndrome (IBS) is a functional disorder of the gastrointestinal tract. A population study showed relatively high prevalence rates of around 10% based on the previous criteria (Rome III) and around 4% according to the latest diagnostic criteria (Rome IV) [1]. The associated medical and economic problems have also been highlighted [2]. The Rome IV criteria [3], currently utilized internationally, define IBS as recurrent abdominal pain occurring on average at least once a week in the last three months, associated with at least two of the following three conditions: (1) defecation-related, (2) change in defecation frequency, or (3) change in stool characteristics (appearance). The pathophysiological characteristics of IBS include (1) abnormalities in gastrointestinal motility, (2) decreased gastrointestinal sensory threshold, and (3) psychological abnormalities, all of which are related to abnormalities in the brain–gut connection. Because IBS a gastrointestinal psychosomatic disorder that is often worsened by stress, a comprehensive approach that addresses its physical and mental aspects is essential [4]. Individuals with IBS exhibit specific cognitive (e.g., bowel performance anxiety, feelings of being out of control, and shame) and behavioral (e.g., avoidance and control behaviors due to fear of abdominal symptoms) responses to their symptoms [5]. Cognitive behavioral therapy (CBT) has been reported to be effective in improving these issues [5, 6].

IBS has previously been reported to be related to the physical and mental health of workers and work-related issues [7]. In addition to the results of population studies, workers with IBS have been reported to have a poor quality of life [8–11], severe sleep-related problems [12], increased burnout [13], and a higher prevalence of psychiatric disorders [14], all of which are associated with their work [15, 16]. Several studies have focused on productivity impairments in workers with IBS, including presenteeism and absenteeism [8, 9, 15, 16]. An association between IBS and shift work has been previously reported, and some meta-analyses have indicated a higher prevalence of IBS among shift workers [12, 17]. Various treatments for IBS, such as medication [18], self-management programs, including CBT [19], and dietary treatment [20], may improve the work productivity of individuals with IBS.

IBS significantly impacts work productivity, which may be related to the perception of abdominal symptoms (e.g., perceived as threatening) and behavioral responses to symptoms (e.g., avoidance of certain situations). Further examination of how occupational stressors, including the work environment, influence the relations among these variables would help develop effective interventions for workers with IBS. Accordingly, we previously examined the interaction of symptom-related cognitive and behavioral measures and occupational stressors on the abdominal symptoms and work productivity of Japanese workers with IBS. We found that maladaptive cognition of abdominal symptoms was associated with stronger abdominal symptoms in low work discretion situations and that maladaptive cognitions, abdominal symptoms, and workload may intensify work productivity losses [21]. In addition, a comparison between groups of workers with IBS after cluster classification by occupational stressors (workload, degree of work discretion, and supervisor and coworker support) [22] showed that symptom-related maladaptive cognitions and anxiety could be more pronounced in situations with low levels of work discretion and ambient support, even in low-load workplaces. Improving these workplace factors may improve anxiety related to abdominal symptoms, which, in turn, would likely improve work productivity.

However, because our previous study was cross-sectional, we were unable to address causal relations between abdominal symptoms or work productivity and the various psychosocial factors associated with them. Therefore, we conducted this longitudinal survey on the effects of IBS and related cognitive-behavioral and occupational stress factors on the abdominal symptoms and productivity impairment among Japanese adult workers one year after an initial evaluation.

## Methods

### Participants

Online surveys were carried out from October 5 to October 20, 2023 (Time 1) and October 5 to October 21, 2024 (Time 2). We included workers aged 20–49 years, excluding those working less than 30 h per week, directors or managers of companies, self-employed individuals, and those with potential organic bowel diseases.

Participants were recruited by Hamon Co. (Yokohama, Japan), a marketing research firm with access to 4,116,490 active members (as of November 4, 2023). Recruitment was persons from all prefectures of Japan, with diverse characteristics in terms of sex and age. Registered members were invited via email to report data using an online platform. After receiving a link and providing informed consent, the participants completed the online survey anonymously. They were briefed on the survey procedures and informed that they could interrupt or terminate the survey at any time without providing a reason. If any item was left unanswered, the questionnaire did not allow participants to proceed to the next page. All participants were rewarded with points provided by Hamon Co., which were redeemable as gift certificates or cash.

The data at Time 1 for this study were partially extracted from the dataset used in our previous cross-sectional study [21]. Overall, 190,562 individuals were ultimately invited to participate in the study via email. The participants were randomly selected from among those aged 20–49 years, based on age data registered with the marketing research firm. A total of 40,001 individuals responded to the initial invitation. Among the respondents, 3,813 declined participation after reading the study description. A screening survey was conducted at Time 1 based on the criteria for IBS (Rome IV diagnostic criteria and red flag items) and the inclusion/exclusion criteria (age, working hours per week, and occupation), was then conducted. Among the respondents, 336 opted out due to unwillingness to answer the survey questions. Additionally, 168 were excluded for not meeting the age inclusion criteria, 1,018 based on occupational exclusion criteria, and 11,660 for not meeting the working hours per week criteria. Finally, 23,006 participants were eligible for further screening. A recruitment invitation was sent to these participants, and the survey was closed once the target sample size had been reached. The required sample size was calculated using G*Power software (latest ver. 3.1.9.7; Heinrich-Heine-Universität Düsseldorf, Düsseldorf, Germany). For the analysis involving individuals with IBS, assuming a medium effect size (*f*² = 0.15), alpha level of 0.05, statistical power of 0.80, and 14 predictors in a multiple regression model, the minimum required sample size was estimated to be 135 participants. Considering the anticipated follow-up rate of approximately 50% for the second survey, we estimated that data from at least 270 individuals with IBS would be required for the first survey (135 × 2 participants). The sample size was also sufficient to meet statistical power requirements for the overall regression analysis with nine predictors, as estimated by G*Power (114 participants for a medium effect size, α = 0.05, power = 0.80). Based on these results and to enhance the robustness of all analyses, we set a target of recruiting at least 300 participants in the IBS group and approximately 1,000 participants in total at Time 1. Of the respondents, 1,062 who did not report any suspected organic bowel disease (329 met the criteria for IBS and 733 did not) were selected for this study, with the age distribution (i.e., the ratio of individuals in each 10-year age group) matched for the groups under consideration. At Time 2, a follow-up survey was conducted with these participants, and responses were obtained from 447.

### Measures

#### Sociodemographic characteristics (Time 1)

We collected sociodemographic information from all participants, such as their sex, age, marital status, annual household income, and work-related information.

#### Lifestyle (Time 1)

Items on tobacco and alcohol consumption were included in the questionnaire because they have been reported to be associated with the presence of IBS [23]. Data on the quantity and quality of sleep were also collected because sleep-related problems in workers have been associated with IBS [24, 25]. These items were obtained from the National Health and Nutrition Survey of the Ministry of Health, Labour and Welfare of Japan [26]. Information regarding recent injuries and illnesses was also obtained.

#### IBS diagnostic criteria (Times 1 and 2)

The Rome IV Diagnostic Questionnaire (R4DQ) [27, 28] was developed to identify for functional gastrointestinal disorders. For this study, only the IBS module was applied to screen for IBS. This module consists of six questions: Q1, the frequency of abdominal pain in the last three months; Q2, abdominal pain related to defecation; Q3, abdominal pain associated with a change in stool frequency; Q4, abdominal pain associated with a change in stool form (appearance); Q5, the onset of abdominal symptoms at least six months before diagnosis; and Q6, the Bristol Stool Scale to define abnormal stools. The criteria for IBS were as follows: (1) Q1 = at least weekly, (2) two or more Q2–Q4 on at least 30% of the occasions, and (3) Q5 = yes. Q6 was used to classify individuals into four subtypes: IBS with predominant constipation, IBS with predominant diarrhea, IBS with mixed bowel habits, and unclassified IBS.

Four red flag items (blood in the stool, awakening due to abdominal pain during night sleep, drastic weight loss, and fever) from the guidelines for IBS of the American Gastroenterological Association were used to distinguish organic bowel diseases. Individuals who reported one or more of these items were excluded from this study.

In the statistical analysis of this study, the presence or absence of IBS at Time 1 was used. However, because it was also necessary to consider whether the participants met the IBS criteria at Time 2, these items were included in the questionnaire for the second survey as well.

#### IBS duration and treatment (Time 1)

We asked how long the abdominal symptoms in the R4DQ section had been present and whether the participants had received treatment for these abdominal symptoms.

#### IBS severity (Times 1 and 2)

The Japanese version of the IBS Severity Index (IBS-SI) [29] was utilized to evaluate the severity of gastrointestinal symptoms. This scale includes five conditions: abdominal pain (two questions), abdominal distension, bowel movements, and quality of life, with a total score ranging from 0 to 500. Based on the IBS-SI score, IBS severity is classified as mild (75–174), moderate (175–299), or severe (300–500) [30].

#### Maladaptive cognition related to abdominal symptoms (Times 1 and 2)

The Japanese version of the Cognitive Scale for Functional Bowel Disorders (CS-FBD) [31, 32] consists of 23 questions that evaluate maladaptive cognition related to abdominal symptoms (e.g. “Pain will never go away,” “Worry about losing control of bowels in public”). Each question is rated on a 7-point scale from 1 (strongly disagree) to 7 (strongly agree).

#### Behavioral response to IBS symptoms (Times 1 and 2)

The Japanese version of the Irritable Bowel Syndrome-Behavioral Responses Questionnaire (IBS-BRQ) [31, 33] consists of 18 questions. Each question is rated on a 7-point scale from 1 (never) to 7 (always). The IBS-BRQ contains questions on behavior obsessed with abdominal symptoms and avoidance of abdominal symptoms or associated difficulties.

#### Absenteeism during the year between time 1 and time 2 (Time 2)

Participants were asked about absenteeism in the past year at Time 2. Three questions were included: absenteeism in the past year, number of days of absenteeism, and absenteeism due to bowel symptoms.

#### Work productivity and activity impairment (Times 1 and 2)

The Work Productivity and Activity Impairment Questionnaire-General Health (WPAI-GH) [34] is a questionnaire used to assess absenteeism, presenteeism, and impairments in work and activities due to health problems during the past seven days. The WPAI-GH contains six questions: Q1 = currently employed, Q2 = hours missed due to health problems, Q3 = hours missed for other reasons, Q4 = hours actually worked, Q5 = the degree of health-affected productivity while working (using a 0-to-10 visual analog scale [VAS]), and Q6 = the degree of health-affected productivity in regular unpaid activities (using the VAS). Four domain scores of the WPAI-GH were calculated and were expressed as percentages by multiplying the following scores by 100: (1) the percentage of work time missed due to health problems = Q2/(Q2 + Q4) for those who were currently employed; (2) the percentage of overall impairment due to health problems = Q5/10 for those who were currently employed and actually worked in the past seven days; (3) the percentage overall work impairment due to health problems = Q2/(Q2 + Q4) + ((1 − Q2/(Q2 + Q4)) × (Q5/10)) for those who were currently employed; (4) the percentage of activity impairment due to health problems = Q6/10 for all respondents [34].

#### Occupational stress factors (Times 1 and 2)

The Brief Job Stress Questionnaire (BJSQ) [35] was developed to evaluate occupational stress in the four weeks prior to the survey. The BJSQ comprises 57 questions covering job stressors (17 questions: e.g., psychological job demands and job control), psychological and physical stress reactions (29 questions), and buffering factors such as social support at work (11 questions). Only five subscales (qualitative and quantitative job overload, job control, and supervisor and coworker support) were used in this study (28 questions). Each question is rated on a 4-point scale from 1 (very true) to 4 (not true at all).

#### Depression (Times 1 and 2)

The Japanese version of the Patient Health Questionnaire-9 (PHQ-9) [36] was used to assess depression symptoms. The PHQ-9 comprises nine questions about symptoms of depression during the past four weeks, with response options ranging from 0 (not at all) to 3 (nearly every day) [37].

#### Anxiety (Times 1 and 2)

The Japanese version of the Generalized Anxiety Disorder-7 (GAD-7) [38] was used to evaluate anxiety symptoms. The seven questions are rated on a scale of 0 (never) to 3 (almost every day). Based on the GAD-7 score, anxiety severity is classified as no anxiety disorder (0–4), mild anxiety (5–9), moderate anxiety (10–14), or severe anxiety (15–21) [39].

#### Somatic symptoms (Times 1 and 2)

The Japanese version of the Somatic Symptom Scale-8 (SSS-8) [40] was used to assess somatic symptom burden. The eight items are rated on a scale of 0 (not at all) to 4 (very much), with the total score ranging from 0 to 32 points [41].

The primary exposure variables in this study were the presence of IBS, its cognitive-behavioral factors related to IBS (as measured by the CS-FBD and IBS-BRQ), and occupational stress factors. The outcome variables were work productivity impairment (measured by the WPAI-GH) and abdominal symptoms (measured by the IBS-SI). Psychological factors including depression (PHQ-9), anxiety (GAD-7), and somatic symptoms (SSS-8) were treated as secondary exposure variables given their potential to affect both outcome variables. These variables were included to study their independent effects, control for potential confounding factors in the regression models, and describe the characteristics of these psychological factors in both the IBS and non-IBS groups.

### Statistical analysis

The *t*-test was used to compare continuous variables of the participants with and without IBS at Time 1. Lifestyle-related items and sociodemographic characteristics at Time 1 according to IBS were compared using the *χ*^2^ test. Comparisons of each psychosomatic and social variable between the groups and time points were performed using two-factor analysis of variance with repeated measures. Using data from the IBS group, the relation between the indices was analyzed by calculating Pearson’s correlation coefficient. Hierarchical multiple regression analysis was used to examine whether IBS-related variables, occupational stress factors, and physical and mental health variables at Time 1 could explain work productivity and abdominal symptoms at Time 2.

For all tests, statistical significance was set at α = 0.05, two-tailed. Statistical analyses were performed using SPSS Statistics version 29.0 (IBM Corp., NY, USA) and R 4.3.2 (R Foundation for Statistical Computing, Vienna, Austria, only for simple slope analysis).

## Results

### Sociodemographic and lifestyle characteristics

We examined whether there were any differences in the psychosocial variables of those who responded to both surveys (*n* = 447) and those who dropped out at Time 2 (*n* = 615). Significantly more women than men did not participate at Time 2 (68.2% vs. 46.8%, *p* < 0.001). Individuals who did not participate at Time 2 were significantly younger and had higher scores on IBS-related variables (*p* < 0.001), sub-scores of the WPAI-GH except for “percent work time missed due to health” (*p* < 0.001), quantitative job overload (*p* = 0.024), the PHQ-9 (*p* < 0.001), the GAD-7 (*p* < 0.001), and the SSS-8 (*p* < 0.001). Upon confirming whether participants met the IBS diagnostic criteria using the R4DQ at Time 2, 75 individuals (65.8%) in the IBS group no longer fully met the criteria. These 75 individuals did not show significant differences in IBS-related variables (IBS-SI, CS-FBD, and IBS-BRQ) compared with those who continued to meet the diagnostic criteria at Time 2. They were thus included in the analysis. Meanwhile, 23 individuals (6.9%) in the non-IBS group met the IBS diagnostic criteria at Time 1, and their data were excluded from the analysis to more accurately assess the effects of IBS, resulting in a final sample of 424 participants (114 in the IBS group and 310 in the non-IBS group). Table 1 shows a comparison of sociodemographic and lifestyle characteristics according to the presence of IBS at Time 1. Women constituted 52.6% of the IBS group and 32.6% of the non-IBS group, indicating a significant difference in sex ratio between the groups. In the IBS group, the duration of symptoms at Time 1 was 9.4 years (standard deviation [SD] = 8.8 years), and 1.4% of the participants were undergoing treatment for abdominal symptoms.

**Table 1:** Comparisons of the sociodemographic and lifestyle indexes at time 1 of the IBS and non-IBS groups

Sociodemographic and lifestyle indexes at Time 1	*N* (%)			Difference between groups		
	Total	IBS group	Non-IBS group	*χ* ^2^	*p*	*φ*
Overall	424	114 (100.0%)	310 (100.0%)			
Sex				14.2	< 0.001	0.183
Male	263	54 (47.4%)	209 (67.4%)			
Female	161	60 (52.6%)	101 (32.6%)			
Academic history				1.9	0.867	0.066
Junior high school	15	4 (3.5%)	11 (3.5%)			
High school	96	25 (21.9%)	71 (22.9%)			
Vocational school	45	12 (10.5%)	33 (10.6%)			
Junior college	17	7 (6.1%)	10 (3.2%)			
University	218	57 (50.0%)	161 (51.9%)			
Graduate school	33	9 (7.9%)	24 (7.7%)			
Marital status				0.3	0.585	0.026
Married	169	43 (37.7%)	126 (40.6%)			
Unmarried	255	71 (62.3%)	184 (59.4%)			
Cohabitation				0.7	0.391	0.042
Yes	303	85 (74.6%)	218 (70.3%)			
No	121	29 (25.4%)	92 (29.7%)			
Raising preschool children				0.2	0.679	0.020
Yes	53	13 (11.4%)	40 (12.9%)			
No	371	101 (88.6%)	270 (87.1%)			
Family caregiving				0.6	0.412	0.039
Yes	17	6 (5.3%)	11 (3.5%)			
No	407	108 (94.7%)	299 (96.5%)			
Annual household income (JPY)				7.8	0.168	0.136
<2.0 million	17	5 (4.4%)	12 (3.9%)			
2.0–3.9 million	121	43 (37.7%)	78 (25.2%)			
4.0–5.9 million	106	24 (21.1%)	82 (26.5%)			
6.0–7.9 million	90	20 (17.5%)	70 (22.6%)			
8.0–9.9 million	37	11 (9.6%)	26 (8.4%)			
10.0 million	53	11 (9.6%)	42 (13.5%)			
Occupation				11.0	0.202	0.161
Management	26	8 (7.0%)	18 (5.8%)			
Profession	42	9 (7.9%)	33 (10.6%)			
Engineering	44	9 (7.9%)	35 (11.3%)			
Clerical job	160	52 (45.6%)	108 (34.8%)			
Production skilled jobs requiring technology	15	1 (0.9%)	14 (4.5%)			
Production skilled jobs operating machinery	21	5 (4.4%)	16 (5.2%)			
Production skilled jobs with many physical tasks	35	12 (10.5%)	23 (7.4%)			
Service job	67	13 (11.4%)	54 (17.4%)			
Other	14	5 (4.4%)	9 (2.9%)			
Work shift				4.0	0.260	0.097
Only day shift	387	100 (87.7%)	287 (92.6%)			
Shift work without night shift	3	2 (1.8%)	1 (0.3%)			
Shift work with night shift	24	8 (7.0%)	16 (5.2%)			
Only night shift	10	4 (3.5%)	6 (1.9%)			
Working hours per week				2.8	0.725	0.082
≥ 30 h to < 40 h	115	35 (30.7%)	80 (25.8%)			
≥ 40 h to < 50 h	242	60 (52.6%)	182 (58.7%)			
≥ 50 h to < 60 h	42	14 (12.3%)	28 (9.0%)			
≥ 60 h to < 65 h	10	2 (1.8%)	8 (2.6%)			
≥ 65 h to < 70 h	4	1 (0.9%)	3 (1.0%)			
≥ 70 h	11	2 (1.8%)	9 (2.9%)			
Period of employment				4.4	0.493	0.102
< 6 months	17	6 (5.3%)	11 (3.5%)			
≥ 6 months to < 1 year	17	5 (4.4%)	12 (3.9%)			
≥ 1 year to < 3 years	53	14 (12.3%)	39 (12.6%)			
≥ 3 years to < 5 years	54	20 (17.5%)	34 (11.0%)			
≥ 5 years to < 10 years	111	27 (23.7%)	84 (27.1%)			
≥ 10 years	172	42 (36.8%)	130 (41.9%)			
Smoking				6.4	0.095	0.123
Daily smoking	96	18 (15.8%)	78 (25.2%)			
Occasional smoking	11	5 (4.4%)	6 (1.9%)			
Former smoker (no use in the past month)	50	12 (10.5%)	38 (12.3%)			
Non-smoker	267	79 (69.3%)	188 (60.6%)			
Frequency of alcohol use				8.4	0.300	0.141
Everyday	48	9 (7.9%)	39 (12.6%)			
5–6 days a week	30	6 (5.3%)	24 (7.7%)			
3–4 days a week	45	10 (8.8%)	35 (11.3%)			
1–2 days a week	80	18 (15.8%)	62 (20.0%)			
1–3 days a month	53	14 (12.3%)	39 (12.6%)			
Rarely use	50	17 (14.9%)	33 (10.6%)			
Stopped using	5	1 (0.9%)	4 (1.3%)			
No use	113	39 (34.2%)	74 (23.9%)			
Amount of alcohol use (pure alcohol)				6.1	0.301	0.141
< 180 mL	89	1 (25.7%)	70 (30.2%)			
≥ 180 mL to < 360 mL	126	28 (37.8%)	98 (42.2%)			
≥ 360 mL to < 540 mL	50	11 (14.9%)	39 (16.8%)			
≥ 540 mL to < 720 mL	20	7 (9.5%)	13 (5.6%)			
≥ 720 mL to < 900 mL	7	3 (4.1%)	4 (1.7%)			
≥ 900 mL	14	6 (8.1%)	8 (3.4%)			
Sleeping hours				6.5	0.264	0.123
< 5 h	39	14 (12.3%)	25 (8.1%)			
≥ 5 h to < 6 h	129	33 (28.9%)	96 (31.0%)			
≥ 6 h to < 7 h	171	44 (38.6%)	127 (41.0%)			
≥ 7 h to < 8 h	73	21 (18.4%)	52 (16.8%)			
* ≥ 8 h to < 9 h*	11	1 (0.9%)	10 (3.2%)			
* ≥ 9 h*	1	1 (0.9%)	0 (0.0%)			
Quality of sleep				4.2	0.238	0.100
Fully	42	9 (7.9%)	33 (10.6%)			
Fairly well	162	37 (32.5%)	125 (40.3%)			
Not much	179	57 (50.0%)	122 (39.4%)			
Not at all	41	11 (9.6%)	30 (9.7%)			

*φ*: 0.100, small; 0.300, medium; 0.600, large

Based on the presence or absence of IBS at Time 1, the participants were classified into the IBS and non-IBS groups

Table 2 shows the difference in absenteeism in the past year assessed at Time 2 of the IBS and non-IBS groups. The proportion of absenteeism and absenteeism due to bowel symptoms was significantly higher in the IBS group than in the non-IBS group. Regarding the number of days of absenteeism, the IBS group had significantly more people absent from work because of health problems for one to six days than the non-IBS group. Despite no significant group differences for the seven or more days category owing to the small numbers in either group, the total number of people in each group who were absent from work for more than seven days was 15.0% in the IBS group and 6.6% in the non-IBS group.

**Table 2:** Absenteeism during the one-year follow-up survey assessed at time 2 among the IBS and non-IBS groups

Items of absenteeism at Time 2	*N* (%)			Difference between groups		
	Total	IBS group	Non-IBS group	*χ* ^2^	*p*	*φ*
Absenteeism in the past year				31.7	< 0.001	0.273
Yes	128	58 (50.9%)	70 (22.6%)			
No	296	56 (49.1%)	240 (77.4%)			
Numbers of days of absenteeism				34.4	< 0.001	0.285
No absenteeism	296	56 (49.1%)	240 (77.4%)			
1–6 days	92	41 (36.0%)	51 (16.5%)			
7–13 days	22	10 (8.8%)	12 (3.9%)			
14–29 days	4	1 (0.9%)	3 (1.0%)			
30–89 days	4	2 (1.8%)	2 (0.6%)			
90–79 days	2	1 (0.9%)	1 (0.3%)			
≥180 days	4	3 (2.6%)	1 (0.3%)			
Absenteeism due to bowel symptoms				33.4	< 0.001	0.281
No absenteeism	296	56 (49.1%)	240 (77.4%)			
Yes	46	24 (21.1%)	22 (7.1%)			
No	82	34 (29.8%)	48 (15.5%)			

Based on the presence or absence of IBS at Time 1, the participants were classified into the IBS and non-IBS groups

### Characteristics of each psychosomatic and social variable at Times 1 and 2 in the IBS and non-IBS groups

Comparisons of each psychosomatic and social variable and time points are shown in Table 3. Significant group effects were observed in the CS-FBD, the IBS-BRQ, sub-scores of the WPAI-GH except percent work time missed due to health, the quantitative job overload, the GAD-7, and the SSS-8 (IBS group > non-IBS group). Significant effects of time were observed in working days a week, “percent activity impairment due to health”, and supervisor and coworker support (Time 1 > Time 2), and “percent work time missed due to health” (Time 1 < Time 2). Significant interactions were found between group and time in qualitative job overload and PHQ-9 scores. In the simple main effect test, the qualitative job overload score (*p* = 0.015) in the IBS group was higher than that in the non-IBS group at Time 1, while the score at Time 1 was higher than that at Time 2 only in the IBS group (*p* = 0.046). The PHQ-9 score was higher in the IBS group than in the non-IBS group at both time points (*p* < 0.001), while the score at Time 1 was higher than that at Time 2 only in the non-IBS group (*p* = 0.021).

**Table 3:** Comparisons of each psychosomatic and social variable between time points and between the IBS and non-IBS groups

	Mean (SD) in IBS group		Mean (SD) in non-IBS group		Effect of IBS			Effect of time			Interaction		
	Time 1	Time 2	Time 1	Time 2	*F*	*p*	*η* ^2^_*p*_	*F*	*p*	*η* ^2^_*p*_	*F*	*p*	*η* ^2^_*p*_
Working days a week	6.0 (0.7)	5.8 (1.2)	6.0 (0.5)	5.9 (1.1)	0.2	0.674	0.000	12.2	0.001	0.028	0.1	0.805	0.000
Teleworking days a month	2.9 (6.5)	2.0 (5.0)	3.1 (6.6)	3.0 (6.4)	1.0	0.311	0.002	2.7	0.101	0.006	1.5	0.215	0.004
IBS-SI	234.5 (78.7)	214.6(101.9)	86.1 (81.8)	82.7 (85.1)	282.0	< 0.001	0.401	7.5	0.007	0.017	3.7	0.054	0.009
CS-FBD	91.3 (28.1)	91.1 (30.5)	55.1 (29.3)	53.1 (27.6)	165.3	< 0.001	0.282	0.8	0.372	0.002	0.5	0.469	0.001
IBS-BRQ	58.4 (22.6)	62.3 (25.0)	38.9 (22.6)	39.0 (21.4)	93.2	< 0.001	0.181	3.4	0.064	0.008	3.2	0.073	0.008
WPAI-GH													
Percent work time missed due to health	2.4 (8.4)	4.4 (14.8)	1.2 (5.6)	2.6 (12.9)	3.0	0.083	0.008	4.0	0.046	0.011	0.1	0.723	0.000
Percent impairment while working due to health	44.8 (26.9)	41.7 (29.4)	18.9 (23.6)	16.8 (23.6)	101.9	< 0.001	0.215	3.3	0.071	0.009	0.1	0.756	0.000
Percent overall work impairment due to health	45.6 (27.3)	42.9 (30.0)	19.7 (24.1)	17.4 (24.6)	98.5	< 0.001	0.209	2.8	0.095	0.007	0.0	0.879	0.000
Percent activity impairment due to health	43.8 (24.7)	42.2 (27.1)	20.5 (24.7)	16.7 (22.1)	110.0	< 0.001	0.209	4.5	0.034	0.011	0.8	0.378	0.002
BJSQ													
Quantitative job overload	8.1 (2.3)	7.9 (2.5)	7.5 (2.4)	7.5 (2.3)	4.1	0.044	0.010	0.4	0.521	0.001	0.4	0.504	0.001
Qualitative job overload	7.9 (2.2)	7.6 (2.2)	7.3 (2.3)	7.4 (2.3)	2.7	0.101	0.006	1.1	0.291	0.003	5.7	0.017	0.013
Job control	7.4 (2.5)	7.3 (2.4)	7.6 (2.1)	7.6 (2.3)	1.6	0.203	0.004	0.3	0.586	0.001	0.1	0.802	0.000
Supervisor support	7.3 (2.7)	6.4 (2.4)	7.6 (2.5)	6.5 (2.4)	0.8	0.385	0.002	79.4	< 0.001	0.160	0.9	0.352	0.002
Coworker support	7.9 (2.8)	7.0 (2.6)	8.0 (2.5)	6.8 (2.4)	0.0	0.906	0.000	70.3	< 0.001	0.144	0.8	0.386	0.002
PHQ-9	9.4 (5.7)	10.1 (6.8)	5.7 (6.4)	5.0 (5.7)	51.8	< 0.001	0.109	0.0	0.893	0.000	6.5	0.011	0.015
GAD-7	7.7 (5.6)	7.6 (6.1)	4.0 (5.1)	3.6 (4.7)	54.4	< 0.001	0.114	1.4	0.235	0.003	0.3	0.617	0.001
SSS-8	12.4 (5.7)	11.5 (6.8)	5.7 (5.9)	5.5 (6.0)	110.8	< 0.001	0.208	3.8	0.051	0.009	1.6	0.207	0.004

IBS-SI: Irritable Bowel Syndrome Severity Index

CS-FBD: Cognitive Scale for Functional Bowel Disorders

IBS-BRQ: Irritable Bowel Syndrome-Behavioral Responses Questionnaire

WPAI-GH: Work Productivity and Activity Impairment Questionnaire-General Health

BJSQ: Brief Job Stress Questionnaire

PHQ-9: Patient Health Questionnaire-9

GAD-7: Generalized Anxiety Disorder-7

SSS-8: Somatic Symptom Scale-8

Based on the presence or absence of IBS at Time 1, the participants were classified into the IBS and non-IBS groups

The results of correlation analysis between each variable at Time 1 and Time 2 in the IBS group (Table 4) indicated that IBS-related variables, the GAD-7, and the SSS-8 in Time 1 were significantly associated with sub-scores of the WPAI-GH except “percent work time missed due to health,” the GAD-7, and the SSS-8 at Time 2. The quantitative job overload score at Time 1 significantly correlated to “percent work time missed due to health” and “percent activity impairment due to health” and the SSS-8 at Time 2. The qualitative job at Time 1 overload score correlated to the IBS-BRQ, “percent activity impairment due to health,” and the SSS-8 at Time 2. The job control score at Time 1 significantly correlated to “percent impairment while working due to health,” “percent overall work impairment due to health,” the PHQ-9, and the GAD-7 at Time 2. Supervisor support and coworker support scores at Time 1 were significantly correlated with the PHQ-9 and GAD-7 at Time 2, and the former score was significantly correlated with the “percent work time missed due to health” score at Time 2. The PHQ-9 at Time 1 significantly correlated to the CS-FBD, the IBS-BRQ, sub-scores of the WPAI-GH except “percent work time missed due to health,” the GAD-7, and the SSS-8 at Time 2.

**Table 4:** Correlation between each variable at time 1 and time 2 in the IBS group

			Time 2									
			IBS-SI	CS-FBD	IBS-BRQ	WPAI-GH				PHQ-9	GAD-7	SSS-8
						Work time missed due to health	Impairment while working due to health	Overall work impairment due to health	Activity impairment due to health			
**Time 1**	IBS-SI	*r*	0.478	0.303	0.255	-0.015	0.294	0.270	0.348	0.187	0.248	0.381
		*p*	< 0.001	0.001	0.006	0.879	0.002	0.005	< 0.001	0.046	0.008	< 0.001
	CS-FBD	*r*	0.347	0.703	0.647	-0.129	0.379	0.361	0.444	0.140	0.232	0.352
		*p*	< 0.001	< 0.001	< 0.001	0.186	< 0.001	< 0.001	< 0.001	0.137	0.013	< 0.001
	IBS-BRQ	*r*	0.385	0.622	0.786	-0.080	0.390	0.372	0.487	0.129	0.246	0.301
		*p*	< 0.001	< 0.001	< 0.001	0.417	< 0.001	< 0.001	< 0.001	0.172	0.008	0.001
	BJSQ											
	Quantitative job overload	*r*	0.035	0.055	0.089	0.231	0.150	0.167	0.208	0.118	0.124	0.222
		*p*	0.710	0.561	0.349	0.017	0.125	0.086	0.026	0.210	0.190	0.017
	Qualitative job overload	*r*	0.069	0.081	0.204	0.119	0.087	0.114	0.217	0.073	0.111	0.237
		*p*	0.465	0.389	0.030	0.224	0.377	0.246	0.020	0.442	0.240	0.011
	Job control	*r*	-0.155	-0.050	0.003	-0.091	-0.206	-0.226	-0.184	-0.257	-0.293	-0.179
		*p*	0.099	0.599	0.975	0.354	0.034	0.020	0.050	0.006	0.002	0.057
	Supervisor support	*r*	0.086	0.026	0.092	-0.193	-0.083	-0.120	-0.028	-0.202	-0.197	-0.036
		*p*	0.362	0.787	0.332	0.048	0.396	0.221	0.771	0.031	0.035	0.700
	Coworker support	*r*	0.180	0.114	0.125	-0.174	0.009	-0.021	0.058	-0.258	-0.142	0.012
		*p*	0.055	0.228	0.186	0.075	0.927	0.832	0.538	0.006	0.133	0.900
	PHQ-9	*r*	0.137	0.200	0.188	0.010	0.295	0.289	0.301	0.627	0.436	0.313
		*p*	0.146	0.033	0.046	0.917	0.002	0.003	0.001	< 0.001	< 0.001	0.001
	GAD-7	*r*	0.210	0.214	0.192	0.021	0.323	0.319	0.316	0.633	0.654	0.373
		*p*	0.025	0.023	0.041	0.832	< 0.001	< 0.001	< 0.001	< 0.001	< 0.001	< 0.001
	SSS-8	*r*	0.310	0.371	0.288	0.076	0.417	0.407	0.454	0.380	0.396	0.704
		*p*	< 0.001	< 0.001	0.002	0.441	< 0.001	< 0.001	< 0.001	< 0.001	< 0.001	< 0.001

IBS-SI: Irritable Bowel Syndrome Severity Index

CS-FBD: Cognitive Scale for Functional Bowel Disorders

IBS-BRQ: Irritable Bowel Syndrome-Behavioral Responses Questionnaire

WPAI-GH: Work Productivity and Activity Impairment Questionnaire-General Health

BJSQ: Brief Job Stress Questionnaire

PHQ-9: Patient Health Questionnaire-9

GAD-7: Generalized Anxiety Disorder-7

SSS-8: Somatic Symptom Scale-8

### Effect of IBS on work productivity impairment due to health problems one year later

In the hierarchical multiple regression analysis, with sample size and multicollinearity considered, the total job overload and total support at work scores were calculated because of the strong correlation between the qualitative and quantitative job overload scores and between the supervisor support and coworker support scores.

Hierarchical multiple regression analysis was conducted, with each WPAI-GH sub-score at Time 2 as the dependent variable, sex and the same variable as the dependent variable at Time 1 (Step 1), and the PHQ-9, the GAD-7, the SSS-8, each occupational stressor (Step 2), and the presence of IBS at Time 1 (Step 3) as explanatory variables, to confirm the effect of IBS on work productivity impairment (Table 5). Regarding the “work time missed due to health problems” score at Time 2, the explained variance score significantly increased from Steps 1 to 2, but the increase was not shown from Steps 2 to 3, and the GAD-7 and the SSS-8 showed significant effects on the dependent variable. Regarding the other sub-scores of the WPAI-GH at Time 2, significant increases in the explained variance scores were observed between the steps. For the “impairment while working due to health” score, the PHQ-9, the GAD-7, the SSS-8, and the presence of IBS indicated significant effects on the dependent variable. Regarding “overall work impairment due to health” scores, the PHQ-9, SSS-8, and presence of IBS showed significant effects on the dependent variable. For the “activity impairment due to health” score, the SSS-8 and the presence of IBS were significantly associated with the dependent variable.

**Table 5:** Effect of IBS on work productivity impairment due to health problems one year later

		Time 2										
		Work time missed due to health			Impairment while working due to health			Overall work impairment due to health			Activity impairment due to health	
		*β*	*p*		*β*	*p*		*β*	*p*		*β*	* p *
**Time 1**	**Step 1**											
	Subscale of WPAI-GH ^a)^	0.050	0.345		0.397	< 0.001		0.398	< 0.001		0.400	< 0.001
	Sex	-0.003	0.957		-0.048	0.267		-0.059	0.177		-0.038	0.336
	**Step 2**											
	PHQ-9	0.188	0.057		0.203	0.009		0.174	0.026		0.076	0.273
	GAD-7	-0.245	0.018		-0.164	0.045		-0.147	0.072		-0.061	0.401
	SSS-8	0.166	0.030		0.162	0.015		0.178	0.007		0.188	0.002
	Job control (BJSQ)	0.013	0.822		-0.086	0.052		-0.060	0.179		-0.054	0.182
	Job overload (BJSQ) ^b)^	0.026	0.643		-0.015	0.737		-0.021	0.632		0.058	0.142
	Support at work (BJSQ) ^c)^	-0.077	0.173		0.029	0.513		0.012	0.794		0.044	0.281
	**Step 3**											
	The presence of IBS	-0.001	0.986		0.162	0.001		0.158	0.001		0.190	< 0.001
	***Step 1*** Adj *R*^2^	0.000			0.334			0.334			0.372	
	Δ*R*^2^	0.005 (*p* = 0.392)			0.338 (*p* < 0.001)			0.338 (*p* < 0.001)			0.375 (*p* < 0.001)	
	***Step 2*** Adj *R*^2^	0.022			0.370			0.366			0.412	
	Δ*R*^2^	0.038 (*p* = 0.028)			0.046 (*p* < 0.001)			0.042 (*p* < 0.001)			0.049 (*p* < 0.001)	
	***Step 3*** Adj *R*^2^	0.019			0.388			0.383			0.439	
	Δ*R*^2^	0.000 (*p* = 0.986)			0.019 (*p* < 0.001)			0.018 (*p* = 0.001)			0.027 (*p* < 0.001)	

The *β* values refer to the results of the analysis using all exploratory variables

a) Subscale of the WPAI-GH consistent with the dependent variable in each analysis

b) Job overload = quantitative job overload + qualitative job overload

c) Support at work = supervisor support + coworker support

WPAI-GH: Work Productivity and Activity Impairment Questionnaire-General Health

BJSQ: Brief Job Stress Questionnaire

PHQ-9: Patient Health Questionnaire-9

GAD-7: Generalized Anxiety Disorder-7

SSS-8: Somatic Symptom Scale-8

### Effect of IBS-related factors on work productivity impairment due to health problems and abdominal symptoms one year later

The results from the hierarchical multiple regression analysis only in the IBS group with each WPAI-GH sub-score at Time 2 as the dependent variable and with the same variable as the dependent variable at Time 1 (Step 1), PHQ-9, GAD-7, SSS-8, IBS-related variables, each occupational stressor (Step 2), and interaction terms between CS-FBD and each occupational stressor (Step 3) as explanatory variables are presented in Table 6. Regarding the “work time missed due to health problems” score at Time 2, no significant increases in explained variance scores were observed between steps, and no explanatory variables were associated with the dependent variable. Regarding the other sub-scores of the WPAI-GH at Time 2, significant increases in explained variance scores were observed between Steps 1 and 2 but not between Steps 2 and 3. For the “overall work impairment due to health” scores, the SSS-8 was significantly associated with the dependent variables. Regarding the “activity impairment due to health” score, no explanatory variables were associated with the dependent variable.

**Table 6:** Effect of IBS-related factors on work productivity impairment due to health problems one year later

		WPAI-GH at Time 2										
		Work time missed due to health			Impairment while working due to health			Overall work impairment due to health			Activity impairment due to health	
		*β*	*p*		*β*	*p*		*β*	*p*		*β*	* p*
**Time 1**	**Step 1**											
	Subscale of WPAI-GH ^a)^	0.110	0.292		0.262	0.020		0.300	0.007		0.149	0.170
	Sex	0.122	0.290		0.009	0.924		0.009	0.926		-0.026	0.768
	**Step 2**											
	PHQ-9	-0.006	0.970		0.090	0.516		0.074	0.590		0.153	0.234
	GAD-7	-0.033	0.856		-0.066	0.659		-0.047	0.751		-0.114	0.406
	SSS-8	0.137	0.285		0.217	0.052		0.231	0.036		0.173	0.098
	IBS-SI	-0.004	0.976		-0.049	0.664		-0.082	0.462		0.071	0.497
	CS-FBD	-0.075	0.625		0.098	0.455		0.108	0.404		0.139	0.240
	IBS-BRQ	-0.041	0.788		0.207	0.106		0.189	0.134		0.217	0.067
	Job control (BJSQ)	0.084	0.502		-0.179	0.085		-0.162	0.114		-0.134	0.164
	Job overload (BJSQ) ^b)^	0.098	0.389		-0.047	0.622		-0.057	0.541		0.091	0.303
	Support at work (BJSQ) ^c)^	-0.178	0.121		0.085	0.367		0.073	0.434		0.127	0.158
	**Step 3**											
	CS-FBD*Job control	-0.088	0.451		-0.168	0.085		-0.154	0.109		-0.109	0.240
	CS-FBD*Job overload	-0.220	0.051		0.020	0.834		0.019	0.838		0.017	0.841
	CS-FBD*Support at work	0.119	0.349		-0.069	0.510		-0.048	0.647		-0.056	0.561
	**Step 1** Adj *R*^2^	0.006			0.221			0.252			0.214	
	Δ*R*^2^	0.026 (*p* = 0.278)			0.237 (*p* < 0.001)			0.267 (*p* < 0.001)			0.228 (*p* < 0.001)	
	**Step 2** Adj *R*^2^	-0.030			0.286			0.314			0.328	
	Δ*R*^2^	0.059 (*p* = 0.770)			0.129 (*p* = 0.0498)			0.123 (*p* = 0.051)			0.166 (*p* = 0.003)	
	***Step 3*** Adj *R*^2^	-0.016			0.304			0.323			0.329	
	Δ*R*^2^	0.043 (*p* = 0.253)			0.037 (*p* = 0.165)			0.028 (*p* = 0.258)			0.019 (*p* = 0.381)	

The *β* values refer to the results of the analysis using all exploratory variables

a) Subscale of the WPAI-GH consistent with the dependent variable in each analysis

b) Job overload = quantitative job overload + qualitative job overload

c) Support at work = supervisor support + coworker support

IBS-SI: Irritable Bowel Syndrome Severity Index

CS-FBD: Cognitive Scale for Functional Bowel Disorders

IBS-BRQ: Irritable Bowel Syndrome-Behavioral Responses Questionnaire

WPAI-GH: Work Productivity and Activity Impairment Questionnaire-General Health

BJSQ: Brief Job Stress Questionnaire

PHQ-9: Patient Health Questionnaire-9

GAD-7: Generalized Anxiety Disorder-7

SSS-8: Somatic Symptom Scale-8

**Fig. 1:**
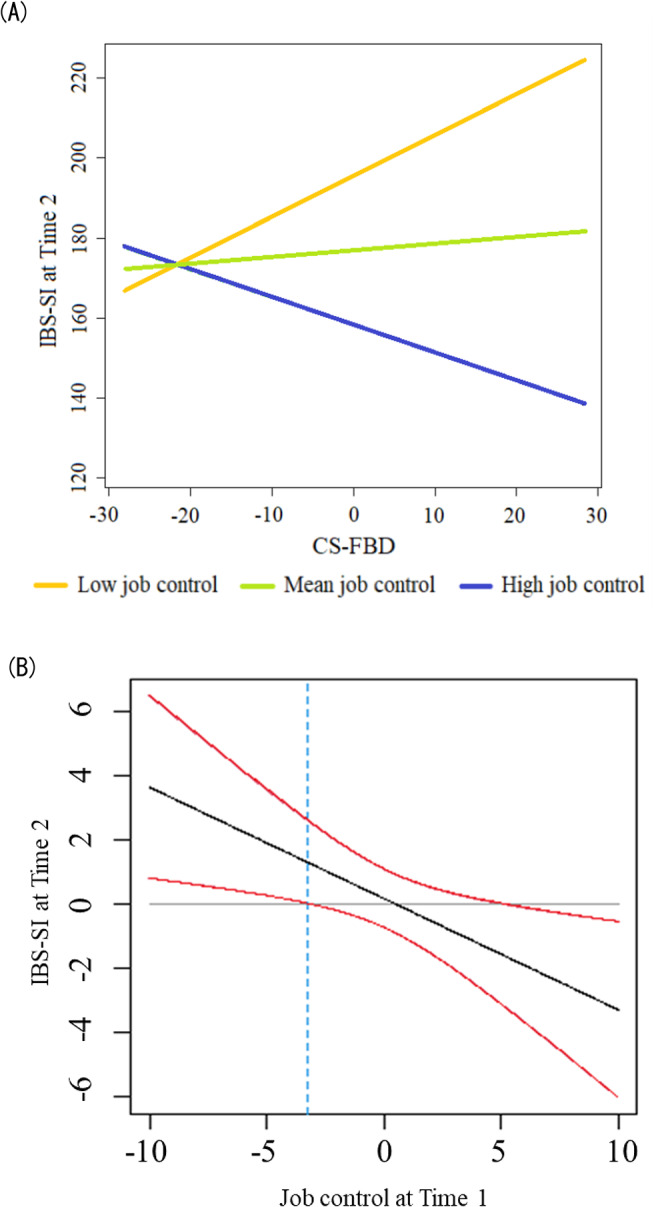
Interaction between job control and maladaptive cognition related to abdominal symptoms on IBS symptoms one year later. (**A**) Simple slopes plot by job control level at Time 1. (**B**) Confidential band (red lines) for the relation between job control at Time 1 and IBS-SI scores at Time 2. IBS: irritable bowel syndrome, IBS-SI: Irritable Bowel Syndrome Severity Index, CS-FBD: Cognitive Scale for Functional Bowel Disorders. The results of the simple slope analysis did not show significant effects when the mean value of the job control score was higher (+ 1 SD, *t* = 1.364, *p* = 0.194) or lower (-1 SD, *t* = 1.901, *p* = 0.078)

Table 7 presents the results from the hierarchical multiple regression analysis in the IBS group with the IBS-SI score at Time 2 as the dependent variable and with the IBS-SI score at Time 1 (Step 1), the PHQ-9, GAD-7, SSS-8, IBS-related variables, each occupational stressor (Step 2), and the interaction terms between CS-FBD and each occupational stressor at Time 1 (Step 3), as explanatory variables. Significant increases in the explained variance scores were observed between steps. The support at work score and the interaction term between the CS-FBD and job control scores showed a significant effect on the dependent variable. In a simple slope analysis for the interaction between the CS-FBD and job control score based on the work by Cohen and Cohen (1983) [42], we created and analyzed data sets in which the mean of the job control score was ± 1 SD. Although the results of the analysis did not show significant effects when the mean value of the job control score was higher (+ 1 SD, *t* = 1.364, *p* = 0.194) or lower (-1 SD, *t* = 1.901, *p* = 0.078), a relation was observed in which the higher the CS-FBD score at Time 1, the higher the IBS-SI score at Time 2, when the mean job control score was lower (Fig. 1).

**Table 7:** Effect of IBS-related factors on abdominal symptoms one year later

		IBS-SI at Time 2							
		Step 1			Step 2			Step 3	
		*B﻿*	*p*		*β*	*p*		*β*	*p*
**Time 1**	**Step 1**								
	IBS-SI	0.464	< 0.001		0.321	0.002		0.241	0.020
	Sex	0.061	0.473		0.087	0.326		0.118	0.179
	**Step 2**								
	PHQ-9				0.043	0.743		0.072	0.576
	GAD-7				-0.026	0.849		-0.086	0.536
	SSS-8				0.106	0.282		0.114	0.241
	CS-FBD				0.029	0.813		0.047	0.693
	IBS-BRQ				0.212	0.081		0.181	0.128
	Job control (BJSQ)				-0.172	0.084		-0.184	0.059
	Job overload (BJSQ) ^a)^				-0.061	0.494		-0.033	0.711
	Support at work (BJSQ) ^b)^				0.259	0.006		0.245	0.008
	**Step 3**								
	CS-FBD*Job control							-0.262	0.006
	CS-FBD*Job overload							-0.013	0.875
	CS-FBD*Support at work							0.175	0.070
	**Step 1** Adj *R*^2^	0.216			0.278			0.315	
	Δ*R*^2^	0.230 (*p* < 0.001)			0.113 (*p* = 0.034)			0.052 (*p* = 0.043)	

a) Job overload = quantitative job overload + qualitative job overload

b) Support at work = supervisor support + coworker support

IBS-SI: Irritable Bowel Syndrome Severity Index

CS-FBD: Cognitive Scale for Functional Bowel Disorders

IBS-BRQ: Irritable Bowel Syndrome-Behavioral Responses Questionnaire

WPAI-GH: Work Productivity and Activity Impairment Questionnaire-General Health

BJSQ: Brief Job Stress Questionnaire

PHQ-9: Patient Health Questionnaire-9

GAD-7: Generalized Anxiety Disorder-7

SSS-8: Somatic Symptom Scale-8

## Discussion

The key results found by this study were the lasting effects of the presence of IBS on presenteeism one year later and the interaction effect between job control and maladaptive cognition related to IBS on abdominal symptoms one year later in workers with IBS.

The presence of IBS was associated with an increase in presenteeism and disruptions to daily life outside of work due to health problems one year later rather than absenteeism. While previous cross-sectional studies have shown a relation between IBS and work productivity, the present study demonstrated that the presence of IBS may reduce overall work productivity in the long term. This result represents a novel finding that has not been previously reported and suggests the need for early intervention for IBS, considering the potential risk of future declines in work productivity. Regarding absenteeism as measured by the WPAI-GH, absenteeism occurred much less frequently than presenteeism, which might explain why no significant effect of IBS on absenteeism was observed in this study. This trend was also observed in our cross-sectional study (*N* = 1062) [21]. Conversely, as shown in Table 2, the presence of absenteeism over one year from baseline (a different measure from the weekly percentage measured by the WPAI for IBS) was significantly higher and more frequent among workers with IBS than among those without IBS. Among those with IBS who experienced absenteeism, 41% reported abdominal symptoms as the cause. Therefore, the possibility that IBS is involved in work productivity through such absences cannot be ruled out. The presence of IBS has been suggested to interfere with activities outside of work, potentially exacerbating the decline in quality of life among workers.

In the analysis focusing solely on participants with IBS, the interaction effect of job control and maladaptive cognition regarding abdominal symptoms on the severity of IBS symptoms one year later was significant. Although no significant differences were detected in the detailed analysis using simple slope tests, maladaptive cognition regarding abdominal symptoms tended to exacerbate abdominal symptoms one year later in situations with low job control. Interventions targeting maladaptive cognition related to abdominal symptoms, along with increasing job control for workers, could contribute to the improvement of abdominal symptoms, and consequently, enhance work productivity. Further investigation is needed to determine how employers can effectively adjust their work environments to improve job control for workers with IBS. To ensure effective care, including a cognitive-behavioral approach, for workers with IBS in their busy lives, it will be necessary to develop, validate, and apply easily accessible tools such as mobile applications recently released to promote physical and mental health. However, this analysis revealed that the amount of support from supervisors and coworkers was associated with the severity of abdominal pain one year later. Although our data could not clarify the reason, the background of having to seek support from those around them may have resulted in a linear relation with the worsening of abdominal symptoms one year later. Correlation analysis showed that higher levels of support were associated with lower levels of anxiety and depression one year later. If observed over a longer period, the relation with improvement in abdominal symptoms might also become apparent.

Among workers with IBS, neither occupational stress factors nor IBS-related variables had a significant effect on work productivity impairment due to health problems one year later. In contrast, general physical symptoms, as measured by the SSS-8 score, had a significant effect. Although correlation analyses showed that both low job control and high scores on IBS-related variables were significantly associated with work productivity impairment at the one-year follow-up, non-abdominal physical symptoms, commonly observed in individuals with IBS, may be stronger predictors of work productivity impairment than these factors. Although IBS-related variables displayed apparent correlations with work productivity one year later, it is possible that lingering physical symptoms such as autonomic nervous system disturbances resulting from the pathophysiology of IBS may directly contribute to reduced work productivity. Future studies that more precisely investigate the interrelations among general physical symptoms, abdominal symptoms, IBS-related cognitive-behavioral factors, job control, and work productivity in workers with IBS are needed to promote a more accurate understanding of the condition.

Fitness to work (FTW) is the process of ensuring that employees perform their duties without compromising their health and safety. In Japan, systems supporting work–treatment balance have been promoted in recent years to strengthen FTW for workers with chronic diseases. Previous studies have shown that individuals receiving support for work–treatment balance perceive higher levels of social support, while those not receiving such support exhibit lower work engagement [43]. While such support is considered essential for workers with IBS, the data from this study showed that only 1.4% of the workers with IBS were undergoing treatment. Although the reasons for this were not clarified by the study data, one possible reason could be the lack of time to receive treatment due to work commitments, thus support for balancing work and treatment could help workers with IBS continue their jobs more comfortably.

In the comparison of each variable between timepoints, working days a week and supervisor and coworker support were decreased and “percent work time missed due to health” score was increased, while abdominal symptoms and “percent activity impairment due to health” score were improved at Time 2. Improvements in daily activity limitations outside of work may also be influenced by changes in environmental factors outside of work. For example, it has been reported worldwide, including Japan, that the COVID-19 pandemic has significantly impacted mental health. In Japan, COVID-19 was reclassified as a Category V infectious disease in May 2023. Social activities are gradually returning to pre-pandemic levels. Previous studies have shown that the deterioration of mental health during a pandemic has gradually improved over time while social isolation and loneliness have remained consistently high [44]. Such psychosocial factors may have influenced the positive or negative changes observed over the one-year period in each variable examined in this study. Some variables showed different patterns of change over time depending on the presence or absence of IBS; qualitative job overload in the IBS group was higher at Time 1 than at Time 2, and PHQ-9 scores in the non-IBS group were higher at Time 1 than at Time 2. The influence of societal changes may vary depending on whether individuals have IBS. For instance, a change in work setting such as the shift from remote work to commuting following the reclassification of COVID-19 in Japan in 2023 might have been more strongly perceived as qualitative job overload among workers with IBS. Alternatively, depressive symptoms that worsened during the pandemic may have improved more smoothly in the non-IBS group than in the IBS group.

In this study, we addressed the association between IBS and work productivity one year later and between occupational stressors and IBS symptoms one year later through a longitudinal investigation that was lacking in previous research on IBS among workers. The results of this study provide important insights into identifying target factors when intervening for workers with IBS. Because occupational stress factors are likely to vary by job type and industry, which factors would help improve in each job type and industry in the future should be examined.

This study had several limitations. First, because the study was conducted using a questionnaire survey, individuals with organic bowel disease might not have been entirely excluded during the IBS screening process. Second, although the IBS and non-IBS groups were matched by age, the sample size was insufficient to achieve a balanced sex ratio. This imbalance occurred because the study rigorously selected individuals with IBS using the Rome IV diagnostic criteria and criteria to exclude organic bowel disease, which resulted in a higher proportion of women in the IBS group. However, previous studies in Japan have shown a higher prevalence of IBS among women, indicating that the sex ratio of the IBS group in this study reflected the actual sex distribution of IBS among Japanese individuals. Third, because the data were collected through an online survey, random sampling was not used, which affected the representativeness of the sample. Fourth, nearly 58% of the participants had dropped out at Time 2. In particular, the number of participants in the IBS group decreased substantially, which might have affected the statistical power of subgroup analyses. Furthermore, those who completed both surveys had significant differences in IBS-related variables, presenteeism, quantitative job overload, depression, anxiety, and general physical symptoms compared with those who did not complete the survey at Time 2. The dropout of participants with poor mental and physical health could have influenced the findings of the study. Upon confirming whether the participants met the IBS diagnostic criteria using the R4DQ during the second survey, it was found that 65.8% of the IBS group no longer fully met the criteria. Meanwhile, 6.9% of the non-IBS group met the IBS diagnostic criteria during the second survey. This may be because many participants with severe IBS-related symptoms dropped out, and many individuals who barely met the diagnostic criteria at Time1 participated in the survey at Time2. These issues could have influenced the lack of significant contributions of occupational stress factors and IBS-related cognitive-behavioral factors to work productivity impairment in the IBS group in this longitudinal study. They may also have affected the nonsignificant findings of the simple slope analysis examining the interaction between job control and maladaptive cognitions related to abdominal symptoms. Fifth, multiple selection steps were taken to winnow participants from the 4,116,490 members of a marketing research firm down to 424. These steps may have introduced a selection bias, meaning that the final sample may not adequately represent the general population. In addition to the likelihood that individuals capable of participating in online surveys were not experiencing severely poor health, our study observed that those who completed both waves of the survey exhibited better physical and mental health indicators than those who dropped out. This suggests that the findings of this study may have been underestimated.

## Conclusion

The presence of IBS was shown to potentially impact the magnitude of productivity loss one year later. Additionally, in situations with low job control, maladaptive cognitions regarding abdominal symptoms tend to exacerbate these symptoms one year later. Combining workplace improvements with psychological approaches, such as CBT, is likely to effectively help workers with IBS maintain their health and work productivity while continuing to work.

## Data Availability

The data that support the findings of this study are available from the corresponding author upon reasonable request.

## Abbreviations

IBS: irritable bowel syndrome
IBS-SI: Irritable Bowel Syndrome Severity Index
CS-FBD: Cognitive Scale for Functional Bowel Disorders
IBS-BRQ: Irritable Bowel Syndrome-Behavioral Responses Questionnaire
WPAI-GH: Work Productivity and Activity Impairment Questionnaire-General Health
BJSQ: Brief Job Stress Questionnaire
PHQ-9: Patient Health Questionnaire-9
GAD-7: Generalized Anxiety Disorder-7
SSS-8: Somatic Symptom Scale-8
