# Correction: Effects of irritable bowel syndrome and related cognitive-behavioral and occupational stress factors on the productivity and abdominal symptoms of Japanese workers: a longitudinal study

**DOI:** 10.1186/s13030-026-00356-0

**Published:** 2026-03-19

**Authors:** Nagisa Sugaya, Shuhei Izawa, Takeshi Sasaki

**Affiliations:** Occupational Stress and Health Management Research Group, National Institute of Occupational Safety and Health, 6-21-1 Nagao, Tama-ku, Kawasaki, Kanagawa 214-8585 Japan

**Correction:** ***BioPsychoSocial Med*** **20, 2 (2026)**

10.1186/s13030-026-00349-z.

Following publication of the original article [1], Fig. 1 image, which was missing from the published version of the article, has been added to the article.

**Fig. 1:**
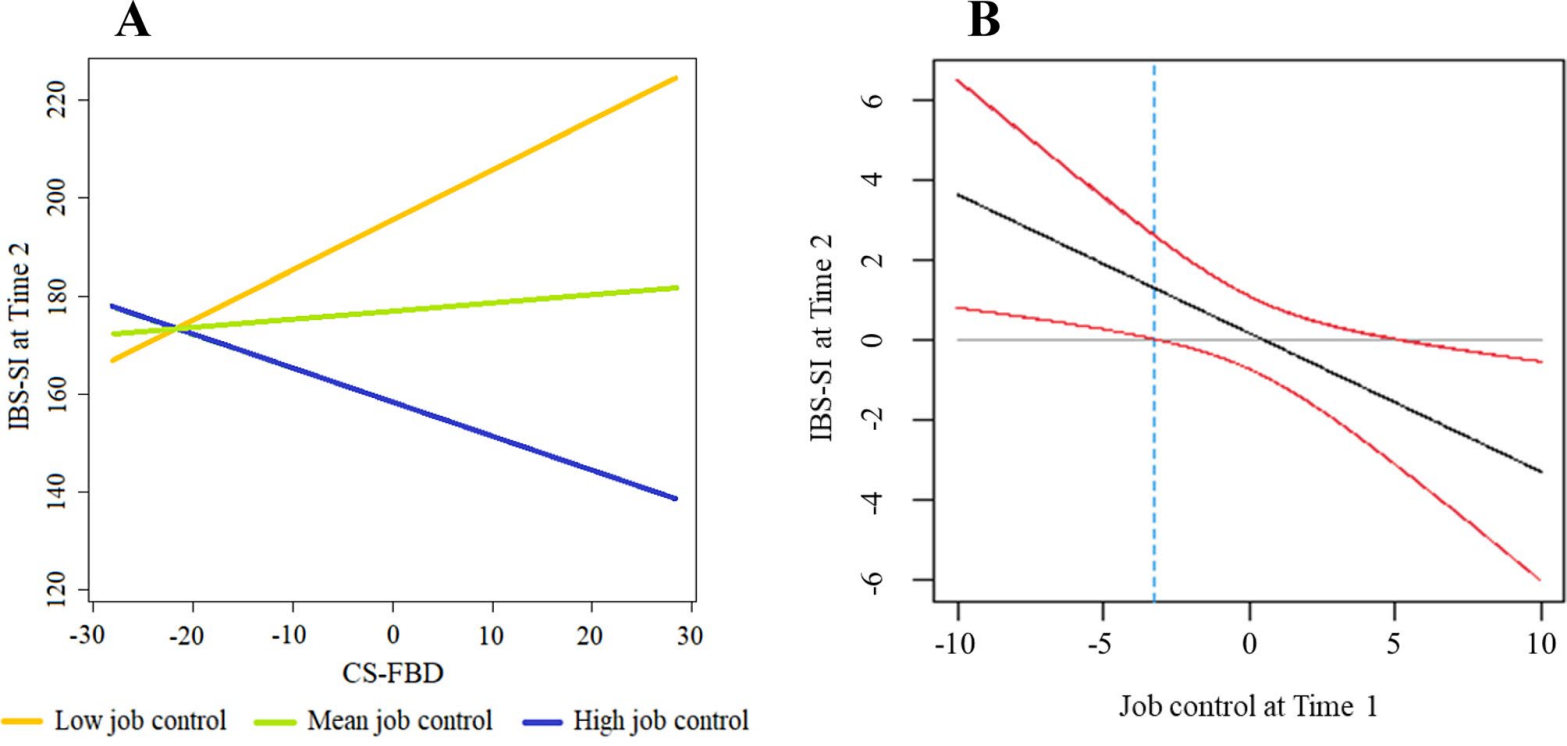
Interaction between job control and maladaptive cognition related to abdominal symptoms on IBS symptoms one year later. (**A**) Simple slopesplot by job control level at Time 1. (**B**) Confidential band (red lines) for the relation between job control at Time 1 and IBS-SI scores at Time 2. IBS: irritable bowel syndrome, IBS-SI: Irritable Bowel Syndrome Severity Index, CS-FBD: Cognitive Scale for Functional Bowel Disorders. The results of the simple slope analysis did not show significant effects when the mean value of the job control score was higher (+1 SD, t=1.364, p=0.194) or lower (-1 SD, t=1.901, p=0.078)

Original article [1] has been updated.
